# Polyacetylene carbon materials: facile preparation using AlCl_3_ catalyst and excellent electrochemical performance for supercapacitors

**DOI:** 10.1039/c9ra01205b

**Published:** 2019-04-16

**Authors:** Tianhang Luo, Xuebing Xu, Maoqiang Jiang, Ying-zhou Lu, Hong Meng, Chun-xi Li

**Affiliations:** State Key Laboratory of Chemical Resource Engineering, Beijing University of Chemical Technology Beijing 100029 P. R. China Licx@mail.buct.edu.cn +86-10-64410308 +86-10-64410308; College of Chemical Engineering, Beijing University of Chemical Technology Beijing 100029 P. R. China; Beijing Key Laboratory of Energy Environmental Catalysis, Beijing University of Chemical Technology Beijing 100029 PR China

## Abstract

Polyacetylene (PA) was synthesized for the first time under mild conditions *via* polymerization of acetylene in *n*-octane with AlCl_3_ as a catalyst, whereby a series of PA-derived carbon materials were obtained. Their composition and structure were characterized and their electrochemical performance was evaluated systematically. It is found that acetylene gas at 1 MPa can polymerize explosively at room temperature under catalysis of AlCl_3_, forming acetylene black-like PA and a great amount of H_2_, while in the presence of *n*-octane solvent, acetylene polymerizes smoothly at higher temperature (30 to 300 °C), forming PA with a H(CH=CH)_*n*_H structure. A series of PA-derived carbon materials are obtained by treating PA with KOH at 800 °C. The as-synthesizzed PA-100–KOH exhibits a high specific surface area (∼2500 m^2^ g^−1^), high specific capacitance (241 F g^−1^ at a current density of 0.1 A g^−1^ and 143 F g^−1^ at 5 A g^−1^), low AC resistance, and good cycling stability with 91.7% maintenance of capacity after 2000 cycles at a current density of 2 A g^−1^. This paper provides a new method for the facile synthesis of PA and a novel carbon source for supercapacitor electrode materials with excellent electrochemical performance and practical application.

## Abstract 2

Polyacetylene (PA) was synthesized for the first time under mild conditions *via* polymerization of acetylene in *n*-octane with AlCl_3_ as a catalyst, whereby a series of PA-derived carbon materials were obtained.

## Graphical abstract

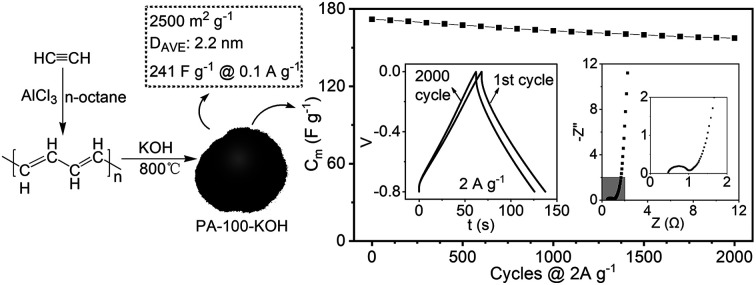

## 1. Introduction

Supercapacitors store energy using either ion adsorption or fast surface redox reactions.^1–4^ Supercapacitors have great potential in the energy storage field^1,5^ due to their fast power delivery and uptake, high power density and environmental friendliness,^6,7^ and the key is to increase the energy density of electrode materials.^3,8,9^ The performance of electrode materials depends on their composition and structure. In this regard, the design and preparation of advanced carbon materials have become a research frontier of supercapacitor materials due to their high specific surface area (SSA),^10^ adjustable pore size distribution^11,12^ and high structure stability.^13^

Advanced carbon materials, including graphene, carbon nanotubes, carbon composites *etc.*,^14,15^ usually exhibit high specific capacitance in the range of 100 to 300 F g^−1^,^2^ and the theoretical specific capacitance of graphene can even reach 550 F g^−1^.^16^ However, their practical application is highly limited by the high cost and least viability for commercial production. In contrast, polyacetylene (PA) might be an ideal precursor of advanced carbon materials considering its rich unsaturated C–C bonds,^17,18^ which bring great potential for its chemical modification and cross-linkage to form carbon materials with desired 3D structure and architecture. Up to date, PA-derived carbon materials have not been used as energy storage materials for supercapacitor to our best knowledge. Traditionally, PA is synthesized using Ziegler–Natta catalyst^19,20^ under harsh conditions, *e.g.* complicated catalyst system, vacuum, cryogenic temperature, and absolute drying,^21,22^ which make it hard to produce in large-scale. In this article, PA was synthesized *via* polymerization of acetylene in *n*-octane under catalysis of AlCl_3_ and heating conditions, and corresponding PA-derived carbon materials were prepared *via* KOH activation at high temperature. Here, PA-derived carbon material was applied as supercapacitor electrode material for the first time, and shows excellent electrochemical performance (∼300 F g^−1^) and promising application prospects.

## 2. Experimental section

### 2.1 Materials and equipment

Acetylene (≥99 wt%) was purchased from Beijing Yongsheng Gas Factory. AlCl_3_ powder (AR, ≥99%) was purchased from Beijing Yongda Chem. Reagent Factory. Nickel foam (10 × 50 × 1.6 in mm, AR, ≥99.8%) was purchased from Taiyuan Liyuan Lithium Electricity Science and Technology Center. Polytetrafluoroethylene suspension (PTFE) (60 wt%) was purchased from Shanghai Aladdin Bio-Chem Technology Co., Ltd. KOH (GR, ≥95%) was purchased from Shanghai Aladdin Bio-Chem Technology Co., Ltd.

The main equipments include oxygen bomb (300 mL, Hebi Hengxin Instrument Co., Ltd.), high-pressure autoclave reactor (50 mL, HC276, Haian County Instrument Factory), air compressor (V-0.25/8, Shanghai Hanli Electrical and Mechanical Equipment Co., Ltd.), tubular reactor (MXG1200-60S, Shanghai Weihang Furnace Co., Ltd.), and electrochemical workstation (CHI660E, Shanghai Chenhua Instrument Co., Ltd.).

### 2.2 Synthesis of PA

Into a 300 mL oxygen bomb (or high pressure autoclave) reactor, 5 g AlCl_3_ and 30 g *n*-octane were added, and the air inside was replaced by three times charging/discharging of acetylene, and finally acetylene was charged to 1 MPa (2.6 g) by the air compressor. The reactor was placed in an oil bath at specified temperature, stirred magnetically, reacted 10 h after the pressure dropped steadily to null, and then cooled to room temperature. The resulting PA was obtained *via* filtration, ethanol washing, and vacuum drying at 100 °C for 2 h. The PA products obtained at reaction temperature of 30, 100, 150, and 300 °C are denoted as PA-30, PA-100, PA-150, and PA-300, respectively. The above experiment was repeated in the absence of solvent at room temperature. When 1 g AlCl_3_ was added to a 50 mL high-pressure autoclave reactor, vacuumed and charged with acetylene to 1 MPa (0.4 g), a blast occurred within 30 seconds after acetylene charging, accompanying with an instant pressure surging. As such, PA black (PA-B) was obtained after the same treatment process above.

### 2.3 Activation of PA

A certain amount of PA was added to the methanol solution of KOH with mass ratio of KOH/PA = 4. The above mixture was irradiated ultrasonically and stirred magnetically for 10 min respectively, and then dried in a rotatory vacuum dryer. The as-treated PA/KOH mixture was placed in a crucible and moved to the tubular furnace, heated to 800 °C at 10 °C min^−1^ and hold for 1 h under argon atmosphere. The furnace was cooled to room temperature naturally. The solid sample was then washed with water to neutral, filtrated, and vacuum dried at 100 °C for 2 h, whereby PA-30–KOH, PA-100–KOH, PA-150–KOH, and PA-300–KOH were obtained. As a contrast, PA-100-800 refers to the thermal treated PA-100 at 800 °C in the absence of KOH following the same procedure above.

### 2.4 Assembly of working electrode

PA carbon material and PTFE were added to ethanol at a mass ratio of 8 : 1, which was then mixed, ultrasonically treated for 10 min, and dried in an oven at 80 °C, yielding a paste. The paste was rolled into a uniform film, and then pressed on one end of the nickel foam current collector with an area of 1 cm^2^. The electrode was prepared by vacuum drying at 100 °C for 4 h. The loading mass of PA carbon material was equal to the mass of nickel foam electrode subtracting the original one.

### 2.5 Measurement of electrochemical performance

The electrochemical performance was tested in a three-electrode system using Hg/HgO as the reference electrode, Pt as counter electrode and 6 mol L^−1^ KOH as electrolyte. All the tests were conducted on a CHI660E electrochemical workstation.

The parameter of galvanostatic charge–discharge (GCD) is as follows: current density 0.1 to 10 A g^−1^, potential range −0.8 to 0 V, test temperature 25 °C. The specific capacitance *C*_m_ (F g^−1^) was calculated by eqn (1).1
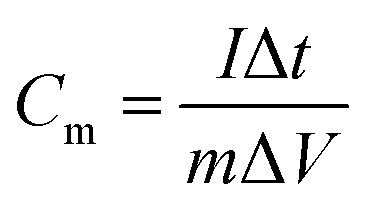
where *I* (A) is the charge–discharge current, *m* (g) is the mass of the PA carbon material, *I*/*m* (A g^−1^) is the charge–discharge current density, Δ*t* (s) is the charge or discharge time and Δ*V* (V) is the potential range of charge–discharge.

The parameter of cyclic voltammetry (CV) is as follows: scan rate 1 to 50 mV s^−1^, potential range −0.8 to 0 V, test temperature 25 °C. The specific capacitance *C*_m_ (F g^−1^) was calculated by eqn (2).2
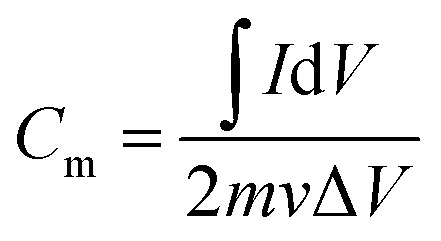
where *m* (g) is the mass of the PA carbon material, *I* (A) is the response current, ∫*I*d*V* is the numerical integration of the CV curve, *v* (mV s^−1^) is the scan rate and Δ*V* (V) is the potential range.

The parameter of electrochemical impedance spectroscopy is as follows: frequency range 10^−2^ to 10^5^ Hz, amplitude 5 mV and test temperature 25 °C.

## 3. Results and discussion

### 3.1 Composition and polymerization mechanism of PA

The elemental analysis results of PA and PA-derived carbon materials are shown in Table 1. As seen from the first three samples, polymerization at higher temperature and in the absence of solvent helps to increase the C-content of PAs. Compared to PA-100 and PA-300, PA-B, as prepared under solvent-free conditions, has the highest C-content (93.7%) and lowest H-content (0.36%), being similar to that of acetylene black (ACET). This indicates the contrast difference of polymerization mechanism of acetylene under different reaction conditions. In fact, both addition polymerization and dehydrogenation polymerization may occur depending on the reaction conditions, as exemplified by the PA synthesis using Ziegler–Natta catalyst^21^ and industrial production of ACET at high temperature,^23^ respectively.

**Table 1:** Elemental analysis results

Sample	N (%)	C (%)	H (%)	S (%)	O (%)
PA-100	0.36	64.14	6.32	0.07	17.59
PA-300	0.06	82.27	4.75	0.21	10.82
PA-B	0.17	93.67	0.36	0.21	1.74
ACET	0.19	100.46	0.32	0.25	1.59
PA-100-800	0.26	79.75	1.20	0.82	9.17
PA-100–KOH	0.39	87.59	1.15	0.30	18.85

The reaction equation is shown in eqn (3) and eqn (4).3
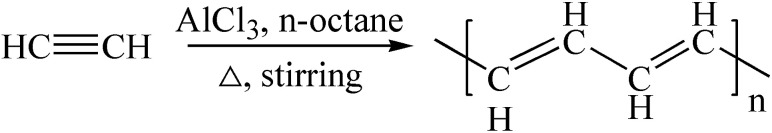
4
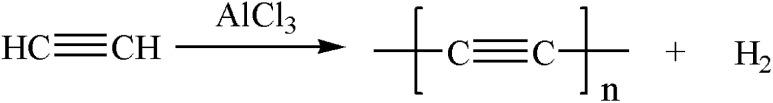


The C-content of PA-100 is 64.1%, which is much lower than the theoretical value of H(CH=CH)_*n*_H, 92.3%. This may be ascribed to its high O-content of 17.59%. If the O-content is deducted, its renormalized C-content is 90.5%, which is close to the theoretical value of 92.3%. Further, the dropped pressure to null after polymerization suggested that the polymerization mechanism of PA-100 follows reaction eqn (3). The high O-content here may be attributed to its high reactivity that is apt to be oxidized partially by air during post treatment and storage processes. This is why PA as conductive polymer is difficult to be used practically.^24,25^

**Fig. 1:**
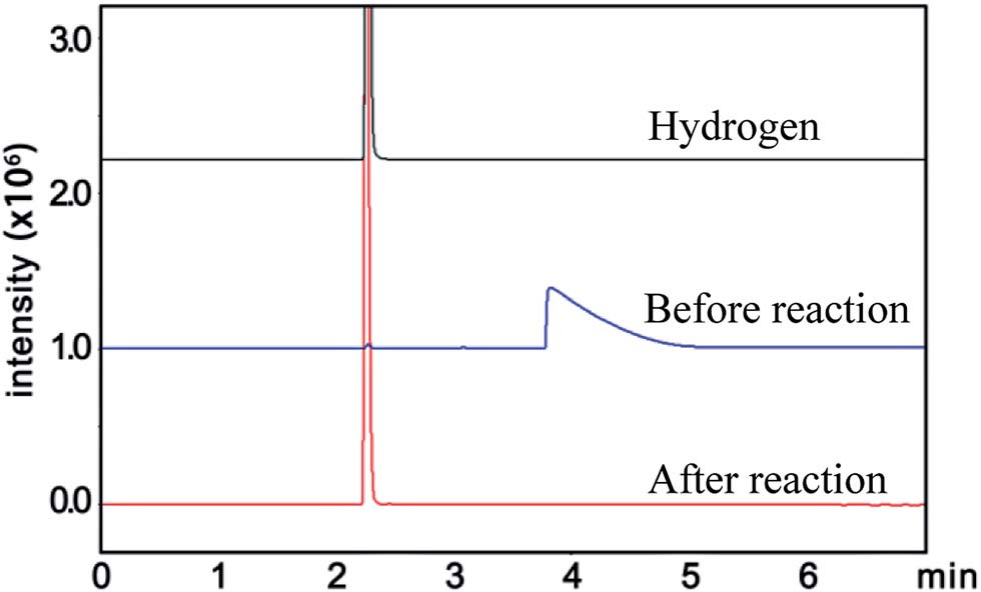
Gas composition before and after the reaction for PA-B.

Similarly, the renormalized C-content of PA-B is 99.2%, which is nearly the same as ACET. In the production process of PA-B, the reaction pressure was found to be increased suddenly from 1 MPa to 1.2 MPa instead of decreased to null. By comparing the gas phase composition before and after polymerization, as shown in Fig. 1, C_2_H_2_ peak disappeared completely along with the appearance of H_2_ peak. This implies that the polymerization reaction proceeds as eqn (4), and the rising pressure may be ascribed to the lower compressibility of H_2_ than C_2_H_2_. In contrast, the renormalized C-content of PA-300 is 94.2%, which is between those of PA-100 and PA-B. Besides, the pressure cannot be decreased to null, being from 1 MPa to 0.6 MPa, which indicates that two polymerization mechanism exist simultaneously for PA-300.

**Fig. 2:**
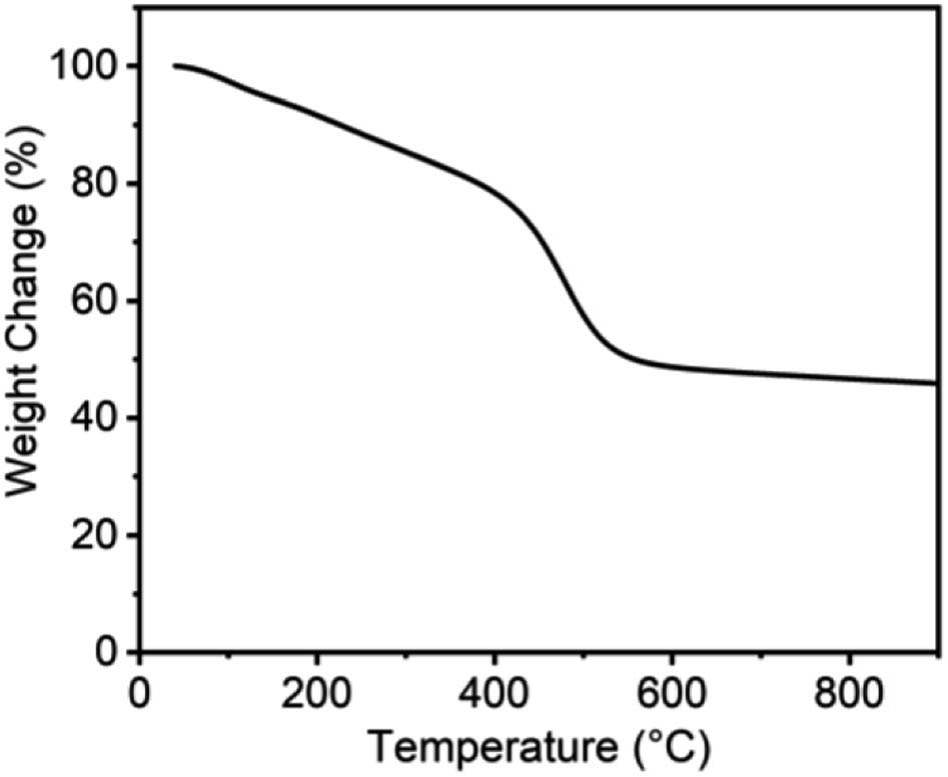
Thermogravimetric analysis of PA-100.

Compared to PA-100, PA-100-800 shows notable increase in renormalized C-content from 90.5% to 97.2% and decrease in H-content from 8.9% to 1.5%. This indicates that high temperature treatment is conducive to dehydrogenation and deoxygenation of carbon materials and accordingly enhancing their C-content, which is consistent with the reported results.^26^ As shown from the thermogravimetric analysis of PA-100 in Fig. 2, there are three distinct mass loss regions, *i.e.* about 25% steady loss between 25 and 400 °C, 28% fast loss between 400 and 550 °C, and 3% of slow loss from 550 to 900 °C, amounting to 55% total mass loss. The first mass loss region may be ascribed to the vaporization of PA-100 oligomers, as justified by the formation of a shining carbon film on the inner side of crucible in the calcination process due to the polymerization of the vaporized oligomers. The drastic weight loss between 400 and 550 °C is probably due to dehydrogenation and deoxygenation of the carbon materials. And the last mass loss may be ascribed to the deep dehydrogenation as well as pyrolysis of the carbon materials. Obviously, the lower yield of PA-100-800 is mainly due to the vaporization of PA-100 oligomers. Compared to PA-100-800, the O-content of PA-100–KOH is doubled to 18.85% due to the carbonation of PA by KOH at high temperature, however, their renormalized C-content is almost the same, being 97.2% and 97.9%, respectively. In effect, it is the thermochemical reaction between KOH and carbon that forms rich microporosity of carbon materials.^27,28^

### 3.2 Characterization of PA carbon materials

**Fig. 3:**
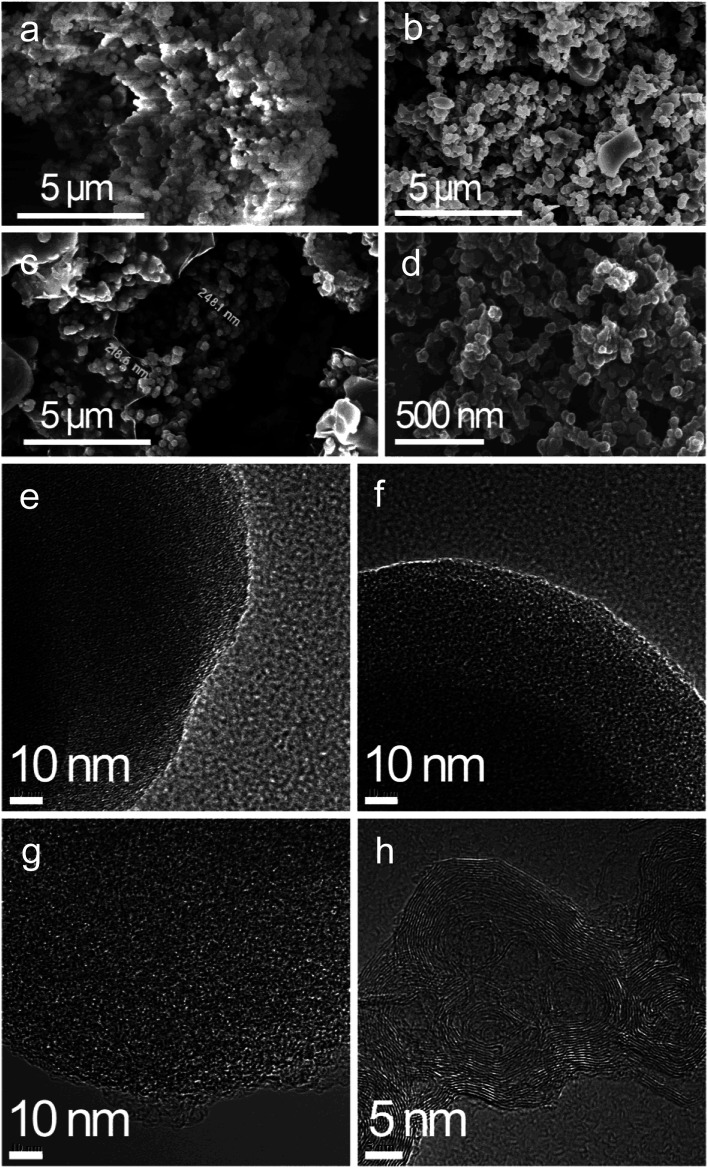
SEM and TEM images of (a) PA-100, (b) PA-100-800, (c) PA-100–KOH, (d) PA-B. (e) PA-100, (f) PA-100-800, (g) PA-100–KOH, (h) PA-B.

As seen from the SEM images of the PA carbon materials in Fig. 3, PA-100 is composed of loose aggregates of submicron particulates, in contrast, PA-100-800 is composed of hard aggregates with larger particle size, which may arise from the high temperature sintering^29^ of the carbon materials with active unsaturated bonds. Compared to PA-100-800, PA-100–KOH is of more serious sintering, forming spherical hard particles of about 230 nm, which may arise from the shaping effect of the molten KOH on the sintering of the PA materials. Compared to PA-100, PA-B is a fluffy rigid aggregate with smaller particle size. As shown from the TEM images, PA-100, PA-100-800 and PA-100–KOH all like a sponge with high cross-linkage, which may arise from thermal crosslinking of the linear PA chains.^24^ In contrast, PA-B shows regular fingerprint structure, a layered graphitic structure, due to the partial graphitization of PA at ultrahigh temperature^23^ as a result of explosive polymerization.

**Fig. 4:**
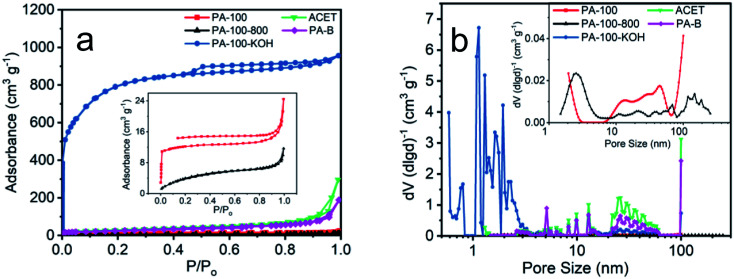
(a) N_2_ adsorption–desorption isotherms and (b) pore size distribution of PA carbon materials.

As shown in Fig. 4a, PA-100–KOH exhibits a type I adsorption isotherm^30^ with highest N_2_-absorbance, total specific area (2530 m^2^ g^−1^) and micro-pore surface area (975 m^2^ g^−1^), which is consistent with its micro-porosity as manifested by the pore size distribution in Fig. 4b. This highlights the importance of KOH activation for the creation of micro- and meso-pores of the PA materials.^27,28^ In contrast, all the remaining PA materials show much lower N_2_-adsorption and specific area, as shown in Fig. 4a and Table 2. Compared to PA-100, PA-100-800 has smaller SSA and pore volume but larger average pore size as a result of high temperature sintering.^29^ Both PA-B and ACET exhibit type II adsorption isotherm^30^ with comparable specific area and similar pore size distribution with predominant meso- and macro-porosity.

**Table 2:** Pore structure parameters of PA carbon materials

Sample	Total *S*_BET_ (m^2^ g^−1^)	Micro *S*_BET_ (m^2^ g^−1^)	*D*_AVE_ (nm)	Total specific pore volume (cm^3^ g^−1^)
PA-100	41	31	3.7	0.038
PA-100–KOH	2530	975	2.3	1.449
PA-100-800	16	14	4.4	0.019
ACET	85	0	6.0	0.147
PA-B	100	0	6.9	0.232

**Fig. 5:**
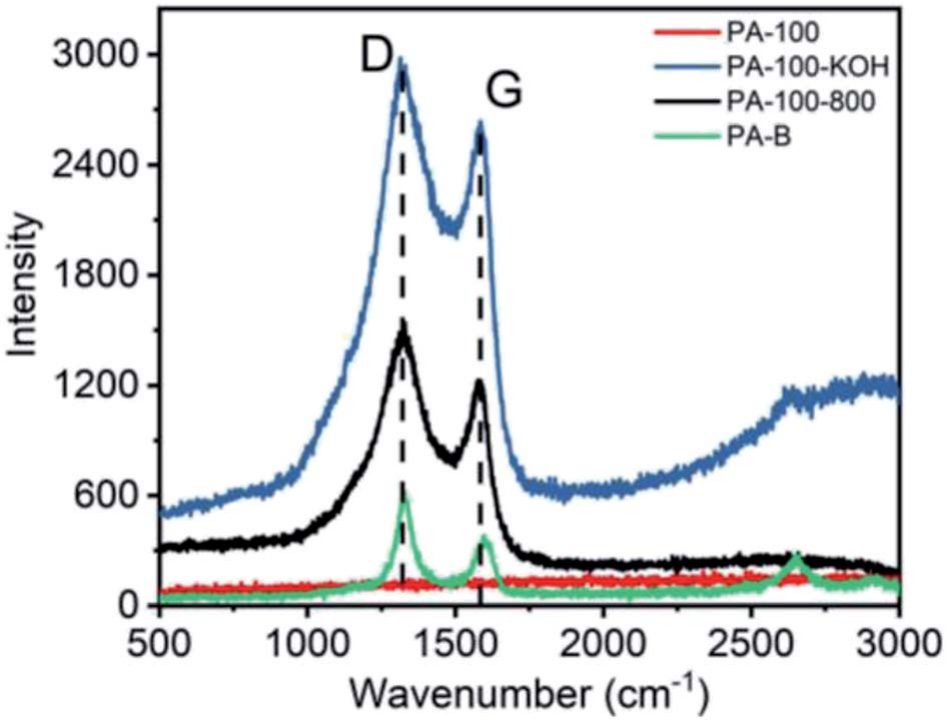
Raman spectra of PA carbon materials.

The Raman spectra of PA materials are shown in Fig. 5. Obviously, all PA materials except PA-100 show D band (∼1330 cm^−1^) and G band (∼1580 cm^−1^), representing sp^3^ and sp^2^ carbon networks, respectively, which is the most prominent feature of graphitic materials.^31^ In contrast, the Raman spectra of PA-100 is not measurable due to its strong fluorescence effect arising from the highly conjugated structure and high O-content.^32^ The intensity ratio (*I*_D_/*I*_G_) of PA-100–KOH, PA-100-800 and PA-B is 6.0, 3.6 and 1.7, respectively, which indicates an increasing graphitization degree since the *I*_D_/*I*_G_ value is reversely proportional to the graphitization degree of carbon materials.^33^ The lower graphitization degree of PA-100–KOH and PA-100-800 is consistent with their TEM images. The disordered D band of PA-B is mainly originated from its edge defects^34^ and disordered stacking,^35^ which coincides with its small particle size and curly graphitic structure as shown in TEM image.

**Fig. 6:**
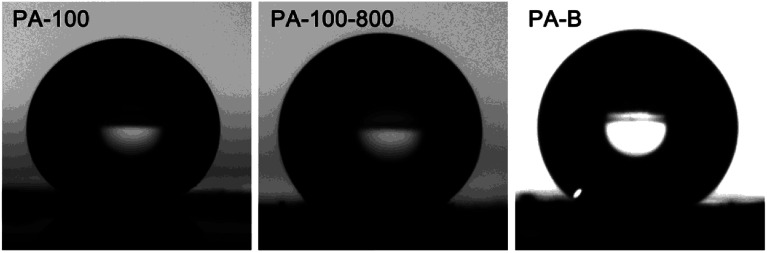
Shape of water droplet on the surface of PA carbon materials.

The water contact angle of PA-100, PA-100-800 and PA-B is determined as 176°, 149° and 149°, respectively, as shown in Fig. 6, which suggests their super hydrophobicity.^36^ In contrast, PA-100–KOH is hydrophilic with non-measurable contact angle.

### 3.3 Electrochemical performance of PA carbon materials

#### 3.3.1 Effect of activation methods on electrochemical performance

The electrochemical performance of PA before and after activation is compared in Table 3. Note worthily, all the pristine PA materials (PA-100, PA-B, ACET) exhibit very poor electrochemical performance with specific capacitance lower than 1 F g^−1^ due to their low SSA^10^ and super-hydrophobicity.^37^ PA-100-800 also shows very poor electrochemical performance, which may be ascribed to its little variation in SSA and hydrophilicity after thermal treatment of PA-100 at 800 °C. In contrast, PA-100–KOH shows very high specific capacity (187.5 F g^−1^) due to its highest SSA and hydrophilicity,^38^ which contrast the modifiability of the PA-100 and the viability of its KOH activation at high temperature. However, the above activation method is less effective for ACET, as manifested from the low electrochemical capacity of ACET–KOH (6.03 F g^−1^), which may be ascribed to its stable structure being formed at very high temperature (∼1800 °C) production process.

**Table 3:** Galvanostatic charge–discharge test (current density 1 A g^−1^)

Electrode	PA-100	PA-100-800	PA-100–KOH	PA-B	ACET	ACET–KOH
*C*_m_ (F g^−1^)	0.06	0.27	187.53	0.53	0.31	6.03

#### 3.3.2 Effect of polymerization temperature on electrochemical performance of carbon materials

**Fig. 7:**
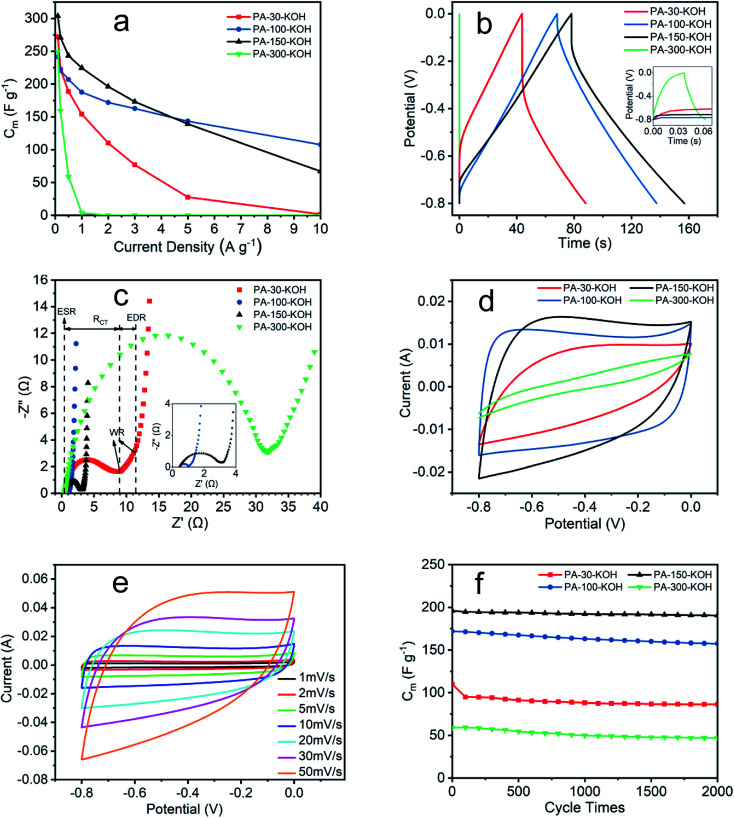
Electrochemical performance of PA carbon materials. (a) and (b) GCD test; (c) Nyquist plot; (d) CV curves at scan rate 10 mV s^−1^; (e) CV curves of PA-100–KOH at different scan rates; (f) cycle life test at current density 2 A g^−1^ except PA-300–KOH at 0.5 A g^−1^.

As observed above, the constitution and structure of the pristine PAs have a determinant role on their modifiability and electrochemical performance of the corresponding PA-derived carbon materials. Thus, the GCD test of PA-derived carbon materials is compared in Fig. 7a and b, as is synthesized at different polymerization temperature and activated by KOH at 800 °C. Obviously, all carbon materials exhibit excellent electrochemical performance with specific capacitance of above 240 F g^−1^ at low current density of 0.1 A g^−1^, see Fig. 7a. However, their specific capacitance declines very differently with the rising current density.^39,40^ The specific capacitance of PA-30–KOH and PA-300–KOH declines most drastically with rising current density, which is solely ascribed to their inappropriate PA precursors (PA-30 and PA-300). The PA precursor polymerized at low temperature has low degree of polymerization,^41^ which leads to a significant decreasing of the specific capacitance at high current density for the resultant carbon material, such as PA-30–KOH. The ACET-like material may be formed in the acetylene polymerization process at 300 °C,^42^ thus the resultant carbon material PA-300–KOH is not suitable for supercapacitor. In contrast, PA-100 has high degree of polymerization and few amount of ACET-like material, and thus the resultant PA-100–KOH shows the best electrochemical performance among them. Moreover, the GCD curve of PA-100–KOH is closer to isosceles triangle, which demonstrates its ideal charge–discharge behavior and suitability as supercapacitor electrode material,^43,44^ see Fig. 7b. In comparison with other carbon materials reported,^45,46^ PA-100–KOH and PA-150–KOH have a relatively high specific capacitance at high current density. The declining performance at high current density may be ascribed to the less ordered structure and wide pore size distribution arising from KOH activation process. In order to develop a well-established structure with even and unique porosity and pore size distribution, templating method^47^ may be a feasible solution. As such, the ion adsorption and desorption become more smooth, which may improve its electrochemical performance at high current density.

The electrochemical impedance of PA carbon materials are shown in Fig. 7c and Table 4. The intersection of Nyquist curve at *x*-axis represents the equivalent series resistance (ESR), which includes the electrolyte solution resistance and contact resistance of electrode material/nickel foam current collector.^48,49^ The charge transfer resistance (*R*_CT_) is given by the diameter of the semicircle,^50^ which is proportional to the resistance at the electrode/electrolyte interface.^49^ The 45° portion namely Warburg region (WR) result from the ion diffusion in electrode,^48^ which represents the equivalent distributed resistance (EDR).^51^ The vertical curve at low frequency indicates an almost ideal capacitor behavior.^43^ The ESR of the four materials are small and similar, which indicates the extremely low resistance of the test system and the excellent assembly consistency of the electrodes. The *R*_CT_ seems positively related to the EDR, revealing the deep correlation between electrode resistance and electrochemical performance from GCD. The lower the resistance, the less the energy loss, the higher stability of the specific capacitance at high current density.^39,40^

**Table 4:** Electrochemical impedance of PA carbon materials

Electrode	ESR (Ω)	*R*_CT_ (Ω)	EDR (Ω)
PA-30–KOH	0.43	8.48	2.42
PA-100–KOH	0.46	0.55	0.22
PA-150–KOH	0.45	2.62	0.19
PA-300–KOH	0.48	31.35	5.21

As shown in Fig. 7d, the CV curves of four materials vary greatly at a scan rate of 10 mV s^−1^. Among them, PA-100–KOH exhibits a nearly rectangular shape, indicating its excellent capacitor behavior and low resistance.^48,51^ The shape distortion of CV curve results from the electrode resistance,^43^ being consistent with previous characterization results. The specific capacitances of PA-30–KOH, PA-100–KOH, PA-150–KOH and PA-300–KOH as calculated from their CV curves are 110, 185, 181 and 15 F g^−1^, respectively. As shown in Fig. 7e, the rectangular shape of CV curves of PA-100–KOH is well remained at scan rate ranging from 1 to 50 mV s^−1^, indicating its good charge propagation and stability.

As shown in Fig. 7f, the specific capacitance of PA-30–KOH, PA-100–KOH, PA-150–KOH and PA-300–KOH remains 78.5%, 91.5%, 97.2% and 79.2%, respectively, after 2000 cycles at current density of 2 A g^−1^ except PA-300–KOH at 0.5 A g^−1^. Overall, the four PA carbon materials exhibit a good cycle stability which is instrumental for supercapacitor electrode materials.^43^

In summary, the specific capacitance of PA-30–KOH and PA-300–KOH decreases significantly at high current density, which may result from the low polymerization degree of PA-30 and ACET-like structure of PA-300. PA-100–KOH shows excellent electrochemical performance with high specific capacitance, low resistance and good cycle stability and thus may be a prospective electrode material for supercapacitor. PA-150–KOH is also a good material but with a higher resistance and worsening electrochemical performance at high current density and scan rate. The electrochemical performance of PA-100–KOH and PA-150–KOH is better than the calcium carbide derived carbon materials.^52,53^

## 4. Conclusions

Under the catalysis of anhydrous AlCl_3_, 1 MPa acetylene gas can polymerize explosively at room temperature, forming ACET-like PA, which is not suitable for commercial production of PA. In contrast, the above polymerization in *n*-octane solvent takes place smoothly at higher temperature (30 to 300 °C) *via* different polymerization mechanism, forming PA with H(CH=CH)_*n*_H structure, which is viable for massive production of PA. The reaction conditions and activation process have great influence on the structure and property of PA and PA-derived carbon materials. All pristine PAs (PA-100, PA-B) are fluffy aggregates of micron particles with rich micro- and/or meso-porosity and high hydrophobicity, and exhibit lower specific area (40 to 100 m^2^ g^−1^) and poor electrochemical performance with specific capacitance lower than 1 F g^−1^. The structure and electrochemical property of the PA-derived carbon materials can be modified greatly by KOH activation at 800 °C, and as-treated PA-100–KOH becomes hydrophilic and exhibits very high SSA (2530 m^2^ g^−1^) and specific capacitance (241 F g^−1^), as well as low resistance and good cycle stability. This study highlights the modifiability of PAs and the viability of KOH activation at high temperature for the preparation of PA-derived carbon materials. In short, this paper provides a new method for facile synthesis of PAs and a new carbon source for supercapacitor electrode materials with excellent electrochemical performance and practical application.

## Conflicts of interest

There are no conflicts to declare.

## Supplementary Material
